# Shared Decision-Making Communication and Prognostic Misunderstanding in the ICU

**DOI:** 10.1001/jamanetworkopen.2024.39715

**Published:** 2024-10-15

**Authors:** Judith B. Vick, Benjamin T. Berger, Peter A. Ubel, Christopher E. Cox, HyunBin You, Jessica E. Ma, Marie C. Haverfield, Bradley G. Hammill, Shannon S. Carson, Catherine L. Hough, Douglas B. White, Deepshikha Charan Ashana

**Affiliations:** 1 Department of Medicine, Duke University, Durham, North Carolina; 2 Durham Center of Innovation to Accelerate Discovery and Practice Transformation, Durham VA Health System, Durham, North Carolina; 3 National Clinician Scholars Program; 4 Department of Population Health Sciences, Duke University, Durham, North Carolina; 5 School of Nursing, Duke University, Durham, North Carolina; 6 Geriatric Research Education Clinical Center, Durham VA Health System, Durham, North Carolina; 7 Department of Communication Studies, San José State University, San José, California; 8 Department of Medicine, University of North Carolina at Chapel Hill; 9 Department of Medicine, Oregon Health & Science University, Portland; 10 Department of Critical Care Medicine, University of Pittsburgh School of Medicine, Pittsburgh, Pennsylvania; 11 Duke-Margolis Center for Health Policy, Duke University, Durham, North Carolina

## Abstract

**Importance:**

Surrogate misunderstanding of patient survival prognosis in the intensive care unit (ICU) is associated with poor patient and surrogate outcomes. Shared decision-making (SDM) may reduce misunderstanding.

**Objective:**

To evaluate the association between SDM-aligned communication and prognostic misunderstanding.

**Design, Setting, and Participants:**

This retrospective cohort study was conducted at 13 medical and surgical ICUs at 5 hospitals in North Carolina, Pennsylvania, and Washington between December 2012 and January 2017. Participants were surrogates of adult patients receiving prolonged mechanical ventilation and ICU physicians. Analysis was performed May to November 2023.

**Exposure:**

SDM-aligned communication during ICU family meetings, defined as the presence of high-quality serious illness communication behaviors aligned with SDM principles.

**Main Outcomes and Measures:**

The primary outcome was postmeeting surrogate prognostic misunderstanding, defined as the absolute difference between the physician’s estimate of survival prognosis and the surrogate’s perception of that estimate (range, 0-100 percentage points). The secondary outcome was postmeeting physician misunderstanding, defined as the absolute difference between a surrogate’s estimate of survival prognosis and the physician’s perception of that estimate (range, 0-100 percentage points). Prognostic misunderstanding of 20 percentage points or greater was considered clinically significant as in prior work.

**Results:**

Of 137 surrogates, most were female (102 [74.5%]), and there were 22 (16.1%) Black surrogates, 107 (78.1%) White surrogates, and 8 surrogates (5.8%) with other race and ethnicity. Of 100 physicians, most were male (64 [64.0%]), with 11 (11.0%) Asian physicians, 4 (4.0%) Black physicians, and 75 (75.0%) White physicians. Median (IQR) surrogate prognostic misunderstanding declined significantly after family meetings (before: 22.0 [10.0 to 40.0] percentage points; after: 15.0 [5.0 to 34.0] percentage points; *P* = .002), but there was no significant change in median (IQR) physician prognostic misunderstanding (before: 12.0 [5.0 to 30.0] percentage points; after: 15.0 [5.0 to 29.0] percentage points; *P* = .99). In adjusted analyses, SDM-aligned communication was not associated with prognostic misunderstanding among surrogates or physicians (surrogates: β = −0.74; 95% CI, −1.81 to 0.32; *P* = .17; physicians: β = −0.51; 95% CI, −1.63 to 0.62; *P* = .38). In a prespecified subgroup analysis of 78 surrogates (56.9%) with clinically significant premeeting prognostic misunderstanding, SDM-aligned communication was associated with reduced surrogate postmeeting prognostic misunderstanding (β = −1.71; 95% CI, −3.09 to −0.34; *P* = .01).

**Conclusions and Relevance:**

In this retrospective cohort study, SDM-aligned communication was not associated with changes in prognostic misunderstanding for all surrogates or physicians, but it was associated with reduced prognostic misunderstanding among surrogates with clinically significant misunderstanding at baseline.

## Key Points

**Question:**

Are shared decision-making (SDM) behaviors in the intensive care unit (ICU) associated with reduced misunderstanding of survival prognosis?

**Findings:**

In this cohort study including 137 ICU family meetings, SDM-aligned communication was not significantly associated with reduced prognostic misunderstanding among surrogates or physicians in the full cohort. However, SDM-aligned communication was associated with reduced surrogate prognostic misunderstanding in a subgroup of surrogates with baseline clinically significant misunderstanding.

**Meaning:**

These findings suggest that for surrogates who have clinically significant prognostic misunderstanding, SDM-aligned communication may reduce prognostic misunderstanding.

## Teaser

This cohort study evaluates whether shared decision-making–aligned communication is associated with prognostic misunderstanding between physicians and surrogates of adult patients in the intensive care unit (ICU).

## Introduction

**Figure 1.**
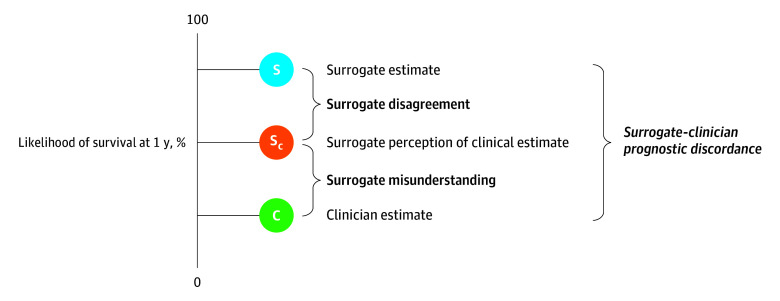
Surrogate Misunderstanding of Physician Estimates of Survival Prognosis This figure illustrates that surrogate-clinician prognostic discordance can come from 2 sources: (1) surrogate misunderstanding of the clinician’s prognostic estimate and (2) surrogate disagreement with their perception of the clinician’s estimate. Surrogate misunderstanding of the clinician’s prognostic estimate is the absolute value of the clinician’s prognostic estimate (C) minus the surrogate’s perception of the clinician’s prognostic estimate (S_c_). Please note that this figure is illustrative only and not based on the data included in our analysis. S indicates surrogate prognostic estimate.

Surrogates often make decisions on behalf of critically ill patients.^1^ Surrogate comprehension of patient survival prognosis can affect these decisions: surrogate prognostic estimates of survival that are overly optimistic compared with clinician estimates are associated with increased use of life-sustaining treatments and reduced quality of life for dying patients.^2,3^ Surrogates’ overly optimistic prognostic estimates of survival have also been associated with clinician-surrogate conflict about treatment decisions, leading to longer lengths of stay in the intensive care unit (ICU) and psychological distress for both parties.^4,5,6,7^ Different prognostic estimates by surrogates and clinicians, or prognostic discordance, may come from 2 sources: (1) surrogate misunderstanding the clinician’s prognostic estimates and (2) surrogate disagreement with the clinician’s prognostic estimate (Figure 1).^2,8,9^ For example, a clinician might estimate that a patient has a 20% chance of survival at 1 year. A surrogate might think that the clinician thinks that the patient has a 50% chance of survival and then disagree with what they think the clinician thinks, stating that they estimate that the patient has a 90% chance of survival at 1 year. In this example, surrogate misunderstanding would be 30 percentage points, surrogate disagreement would be 40 percentage points, and surrogate-clinician prognostic discordance would be 70 percentage points. Disagreement may reflect differing values (eg, intentional optimism to maintain hope), personal knowledge of a patient (eg, familiarity with their personality), and beliefs (eg, religious beliefs) among surrogates and clinicians.^10,11^ While disagreements based on differing values or beliefs may always be present, reducing misunderstanding is a clinical responsibility and a key target of high-quality communication.

Prior work has demonstrated that surrogates frequently misunderstand clinicians’ estimates of survival prognosis.^2^ To reduce this misunderstanding, best practices for clinicians include communicating without the use of jargon or euphemism, responding directly to inquiries regarding a patient’s prognosis, and assessing for understanding.^8,12,13,14^ Shared decision-making (SDM), defined as “a collaborative process that allows patients, or their surrogates, and clinicians to make health care decisions together, taking into account the best scientific evidence available, as well as the patient’s values, goals, and preferences,”^14^ may also lead to reduced surrogate misunderstanding of clinician prognostic estimates due to its focus on collaboration.^9^ SDM’s emphasis on clinicians and patients or surrogates working together may be assumed to improve both surrogate misunderstanding of clinician prognostic estimate (Figure 1) and clinician misunderstanding of surrogate prognostic estimate (eFigure 1 in Supplement 1). No study, however, has empirically examined the association between SDM and misunderstanding of survival prognosis.

The current study aims to address this knowledge gap. While there are no well-validated measures of SDM within the ICU, prior work has identified specific communication behaviors in the context of critical illness that are aligned with SDM (hereafter referred to as SDM-aligned communication) and are associated with the delivery of goal-concordant care.^15,16^ Using audio-recorded ICU family meetings and surrogate and physician estimates of survival prognosis, our objective was to test the hypothesis that increased use of SDM-aligned communication in these family meetings would be associated with (1) reduced surrogate misunderstanding of physician estimates of survival prognosis (hereafter, surrogate prognostic misunderstanding) and (2) reduced physician misunderstanding of surrogate estimates of survival prognosis (hereafter, physician prognostic misunderstanding).

## Methods

This study was approved by the Duke Health institutional review board. We adhered to the Strengthening the Reporting of Observational Studies in Epidemiology (STROBE) guidelines in this manuscript.^17^

### Data Source

We performed a secondary analysis of data from a randomized clinical trial of a decision support tool about prolonged mechanical ventilation (NCT01751061).^18^ As described in full elsewhere, the trial was conducted in 13 ICUs at 5 hospitals in North Carolina, Pennsylvania, and Washington between December 2012 and January 2017. Eligible participants included the surrogate decision-makers of incapacitated adult patients who were mechanically ventilated for at least 10 days and for whom death or liberation from the ventilator was not expected within 24 hours of enrollment. Additional details of inclusion and exclusion criteria have previously been published.^18^ All participants gave written informed consent. Baseline characteristics, including demographics (including age, sex, race and ethnicity) and surrogate health literacy (measured by a previously validated 3-item scale), were self-reported by interview.^19^ Racial and ethnic groups included American Indian or Alaska Native, Asian, Black, Native Hawaiian or Other Pacific Islander, and White. American Indian or Alaska Native and Native Hawaiian or Other Pacific Islander individuals were combined with those selecting multiple groups or other into the other category. Unscripted family meetings with surrogates and ICU physicians from both intervention and nonintervention groups were conducted on day 2 after enrollment.^18^

### Exposure

From 146 audio-recorded and transcribed family meetings, we purposively sampled 137 that had complete recordings and transcripts. Summative content analysis had previously been performed using these transcripts to quantify SDM-aligned communication behaviors in the meetings.^20^ These SDM-aligned communication behaviors had been identified in earlier work as best practice communication behaviors within a framework of SDM and found to be associated with goal-concordant care delivery during a simulated clinical scenario, coinciding with the ultimate purpose of SDM to “make treatment decisions that are medically appropriate and consistent with the patient’s values, goals, and preferences.”^9^ Coders of SDM-aligned communication behaviors were blinded to the outcomes of interest in the current analysis.

In the current study, we defined our primary exposure as SDM-aligned communication, calculated by summing 13 SDM-aligned communication behaviors in the meetings with a range of 0 (fewest behaviors) to 13 (most behaviors) (eTable in Supplement 1). While a rigorously validated observer-based measure of SDM in the ICU context does not exist, our measure of SDM-aligned communication overlaps with the essential elements of SDM (information exchange, deliberation, and making a treatment decision) as defined by consensus of professional critical care societies (eTable in Supplement 1).^9^ Our measure of SDM-aligned communication also includes elements conducive to SDM in critical illness specifically. For example, it includes an item related to spiritual support, which may be more salient for decisions in the ICU than in other contexts.^21,22^

### Outcomes

Surveys to both physicians and surrogates were administered immediately before and immediately after each family meeting. Surrogates were asked both their own estimate of the patient’s survival prognosis (“What do you think is the chance that your loved one will survive for at least one year from now if the current treatment is continued?”) and their perception of the physician’s estimate (“What do you think the patient’s ICU physician believes is the chance your loved one will survive for at least one year from now?”) immediately before and immediately after the family meeting. Physicians were also asked their own estimate of the patient’s survival prognosis and their perception of the surrogate’s estimate both before and after the family meeting.

The primary outcome was postmeeting surrogate misunderstanding of physician prognostic estimate, defined as the absolute value of the difference between the physician’s prognostic estimate after the family meeting and the surrogate’s perception of that estimate (Figure 1). The secondary outcome was postmeeting physician misunderstanding of surrogate prognostic estimate, defined as the absolute value of the difference between the surrogate’s prognostic estimate after the family meeting and the physician’s perception of that estimate (eFigure 1 in Supplement 1).

### Statistical Analysis

We used Wilcoxon signed-rank tests to compare surrogate and physician perceptions of and actual prognostic estimates and prognostic misunderstanding both before and after family meetings.^23^ To determine the association of SDM-aligned communication with both outcomes, we used multilevel mixed-effects linear regression to account for physician clustering (ie, some clinicians conducted more than 1 family meeting). We created directed acyclic graphs (DAGs) in DAGitty to conceptually identify potential confounding variables (eFigure 2 in Supplement 1).^24^ In our multivariable analyses, we adjusted for the confounding variables identified in our DAGs: for surrogate misunderstanding, we adjusted for premeeting prognostic misunderstanding, surrogate trial group (intervention or control), and surrogate health literacy. For physician misunderstanding, we adjusted for premeeting prognostic misunderstanding and surrogate trial group (intervention or control).

We performed several prespecified secondary analyses. We hypothesized that some components of SDM-aligned communication may be more important than others in their association with postmeeting surrogate misunderstanding. To assess this, we performed the following 2 sensitivity analyses. First, we modified the exposure to include only information-sharing behaviors from the full list of SDM-aligned behaviors (SDM information): providing purpose of the visit, discussing prognosis, and using the term death. The rationale was that information exchange, on face value, would most likely be related to understanding. Second, we modified the exposure to include only SDM-aligned behaviors most directly related to prognostic communication (SDM prognosis): discussing prognosis, assessing understanding, asking if family had questions, and using the word death. Our rationale was that these elements were on face value most directly related to prognostic misunderstanding. Finally, we conducted a subgroup analysis of surrogate misunderstanding restricted to cases with premeeting surrogate misunderstanding of 20 percentage points or greater.^10,11^ We reasoned that SDM-aligned communication may only reduce prognostic misunderstanding in cases with greater preexisting surrogate misunderstanding. We chose a cutoff of premeeting surrogate misunderstanding of 20 percentage points or greater based on prior work demonstrating that patients’ willingness to continue life-sustaining treatments declined substantially when their survival prognosis decreased by 20 percentage points.^11,25^ Premeeting surrogate misunderstanding was not included as a confounder in this model because we already restricted to cases with clinically significant premeeting surrogate misunderstanding. We chose a subgroup analysis approach rather than effect modification given our interest in examining SDM-aligned communication within a clinically important subgroup, rather than comparing effects of SDM-aligned communication across different subgroups.

All analyses were performed with Stata SE version 17.0 for Mac (StataCorp) and used complete case analysis. No statistical power calculations were calculated for this study as a secondary analysis of trial data. Statistical significance was defined as a 2-sided *P* ≤ .05. This was not adjusted for multiple testing; therefore, sensitivity and subgroup analyses should be considered exploratory.

## Results

### Demographic Characteristics

Demographic characteristics of participating patients, surrogates, and physicians appear in the Table.^26^ Of 137 patients, a minority were female (48 [35.0%]), with 24 (17.5%) Black patients, 106 (77.4%) White patients, and 7 patients (5.1%) with other race and ethnicity. Of 137 surrogates, most were female (102 [74.5%]), with 22 (16.1%) Black surrogates, 107 (78.1%) White surrogates, and 8 surrogates (5.8%) with other race and ethnicity. Surrogate relationship to the patient was as follows: spouse or partner (64 [46.7%]), parent (31 [22.6%]), adult child (25 [18.2%]), sibling (14 [10.2%]), and other family (3 [2.2%]). On average, health literacy was high among surrogates (median [IQR] 5.0 [4.0-6.0], on a 3-item health literacy scale ranging from 3 to 15, with smaller values indicating higher health literacy).^19^ The 137 family meetings were led by 100 unique physicians. Physicians were mostly male (64 [64.0%]), with 11 (11.0%) Asian physicians, 4 (4.0%) Black physicians, and 75 (75.0%) White physicians.

**Table.** Participant Characteristics and Prognostic Estimates, Perceptions, and Misunderstanding

Characteristic	Participants, No. (%)		
	Patients (n = 137)	Surrogates (n = 137)	Physicians (n = 100)
Demographic characteristic			
Age, median (IQR), y	54.0 (40.0-64.0)	52.0 (43.0-61.0)^a^	35.0 (32.0-42.0)
Sex			
Female	48 (35.0)	102 (74.5)	36 (36.0)
Male	89 (65.0)	35 (25.5)	64 (64.0)
APACHE II score, median (IQR)	23.0 (18.0-28.0)	NA	NA
In-hospital mortality	38 (27.7)	NA	NA
Race			
Asian	0	0	11 (11.0)
Black	24 (17.5)	22 (16.1)	4 (4.0)
White	106 (77.4)	107 (78.1)	75 (75.0)
Other^b^	7 (5.1)	8 (5.8)	10 (10.0)
Randomized to intervention	66 (48.2)	66 (48.2)	NA
Estimates of survival prognosis, median (IQR), %			
Premeeting prognostic estimate	NA	90.0 (54.0-100.0)^c^	58.0 (23.0-75.0)^d^
Postmeeting prognostic estimate	NA	89.5 (50.0-100.0)^e^	60.0 (20.0-78.0)^f^
Perceptions, median (IQR), %			
Premeeting perception of other party’s prognostic estimate	NA	75.0 (50.0-97.0)^c^	80.0 (53.0-95.0)^g^
Postmeeting perception of other party’s prognostic estimate	NA	70.0 (26.0-98.5)^e^	75.0 (50.0-91.0)^h^
Misunderstanding, median (IQR), percentage points			
Premeeting misunderstanding of other party’s prognostic estimate	NA	22.0 (10.0-40.0)^c^	12.0 (5.0-30.0)^i^
Postmeeting misunderstanding of other party’s prognostic estimate	NA	15.0 (5.0-34.0)^j^	15.0 (5.0-29.0)^k^
Premeeting prognostic misunderstanding ≥20 percentage points, No. (%)	NA	78 (56.9)^c^	57 (44.2)^i^
Postmeeting prognostic misunderstanding ≥20 percentage points, No. (%)	NA	55 (42.3)^j^	53 (40.5)^k^

Abbreviations: APACHE, Acute Physiology and Chronic Health Evaluation; NA, not applicable.

^a^ Data available for 135 surrogates.

^b^ Other race included American Indian or Alaskan Native individuals, Native Hawaiian or Other Pacific Islander individuals, individuals reporting multiple races, and those who self-reported other race and ethnicity.

^c^ Data available for 137 surrogates.

^d^ A total of 137 estimates by 100 unique physicians.

^e^ Data available for 132 surrogates.

^f^ A total of 134 estimates by 99 unique physicians.

^g^ A total of 129 perceptions by 97 unique physicians.

^h^ A total of 135 perceptions by 99 unique physicians.

^i^ Data available for 129 cases.

^j^ Data available for 130 surrogates.

^k^ Data available for 131 cases.

### SDM-Aligned Communication

**Figure 2.**
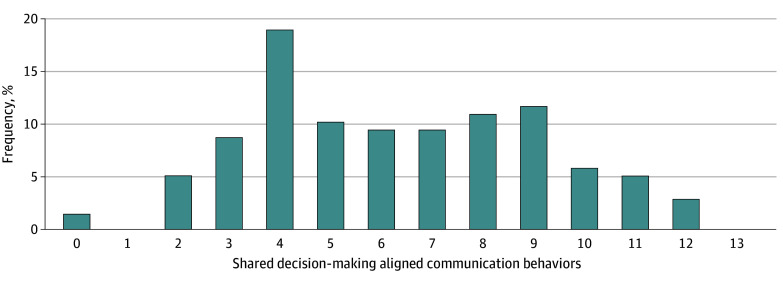
Shared Decision-Making–Aligned Communication Behaviors Shared decision-making–aligned communication behaviors were identified in a prior summative content analysis using a codebook adapted from a shared decision-making–based scoring schema that found that these communication behaviors were associated with goal-concordant care delivery during a simulated clinical scenario involving a critically ill patient.^15^

During the family meetings, the median (IQR) number of SDM-aligned communication behaviors was 6 (4-9; range, 0-12). The distribution of SDM-aligned behaviors appears in Figure 2.

### Estimates, Perceptions, and Misunderstandings of Survival Prognosis

**Figure 3.**
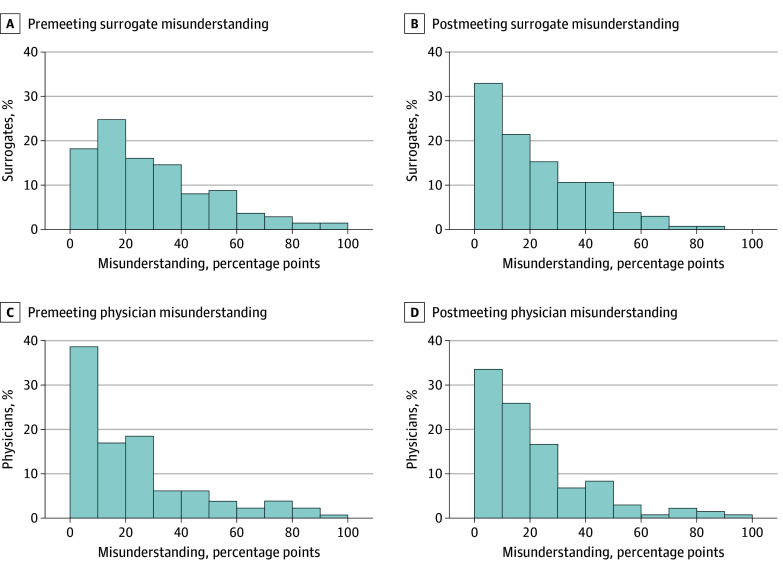
Premeeting and Postmeeting Surrogate and Physician Misunderstanding of Other’s Prognostic Estimate A and B, Median surrogate misunderstanding decreased by 7.0 percentage points. A Wilcox signed rank test showed that there was a significant difference (Z = 3.07; *P* = .002) between premeeting surrogate misunderstanding and postmeeting surrogate misunderstanding. C and D, Median physician misunderstanding increased by 3.0 percentage points. A Wilcox signed rank test showed that there was not a significant difference (Z = −0.02; *P* = .99) between premeeting physician misunderstanding and postmeeting physician misunderstanding.

Overall, surrogates perceived physicians’ prognostic estimates to be significantly higher than the physicians’ actual prognostic estimates both before family meetings (median [IQR], 75.0% [50.0%-97.0%] vs 58.0% [23.0%-75.0%]; *P* < .001) and after family meetings (median [IQR], 70.0% [26.0%-98.5%] vs 60.0% [20.0%-78.0%]; *P* < .001) (Table). Surrogate misunderstanding of physician prognostic estimates declined significantly after family meetings (median [IQR], before: 22.0 [10.0-40.0] percentage points; after: 15.0 [5.0-34.0] percentage points; *P* = .002) (Figure 3A and B). However, many surrogates (55 [42.3%]) still misunderstood the physician’s prognostic estimate by at least 20 percentage points after family meetings. eFigure 3 in Supplement 1 portrays changes in misunderstanding for each surrogate from before to after the family meeting.

Before family meetings, physicians perceived surrogates’ prognostic estimates to be significantly lower than surrogates’ actual prognostic estimates (median [IQR], 80.0% [53.0%-95.0%] vs 90.0% [54.0%-100.0%]; *P* = .04). After family meetings, physicians’ perceptions of surrogates’ prognostic estimates were lower than surrogates’ actual prognostic estimates, but the difference was not statistically significant (median [IQR], 75.0% [50.0%-91.0%] vs 89.5% [50.0%-100.0%]; *P* = .10) (Table).

Physicians’ misunderstanding of surrogate prognostic estimates did not significantly change after the family meeting (median IQR, before: 12.0 [5.0-30.0] percentage points; after: 15.0 [5.0-29.0] percentage points; *P* = .99) (Figure 3C and D). In more than one-third of instances, physicians (53 [40.5%]) misunderstood surrogates’ prognostic estimates by at least 20 percentage points after family meetings. Missingness rates of estimate, perception, and misunderstanding variables are noted at the bottom of Table.

### SDM-Aligned Communication and Prognostic Misunderstanding

**Figure 4.**
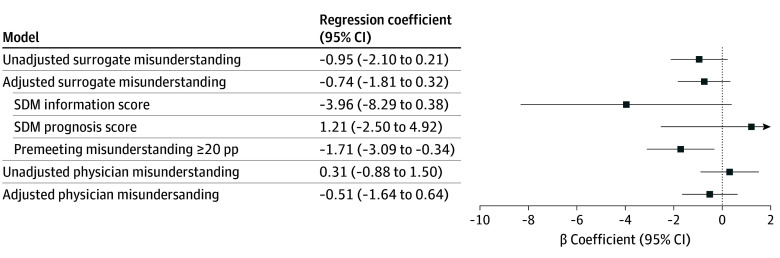
Association of Shared Decision-Making (SDM)–Aligned Communication and Surrogate and Physician Prognostic Misunderstanding pp Indicates percentage point.

There were no significant associations between SDM-aligned communication and surrogate prognostic misunderstanding, in either unadjusted or adjusted models (unadjusted: β = −0.95; 95% CI, −2.10-0.21; *P* = .11; adjusted: β = −0.74; 95% CI, −1.81-0.32; *P* = .17) (Figure 4). In the unadjusted model, every additional SDM-aligned communication behavior was associated with a 0.95–percentage point decrease in surrogate misunderstanding; in the adjusted model, every additional SDM-aligned communication behavior was associated with a 1.81–percentage point decrease in surrogate misunderstanding, but these associations were not statistically significant. In the adjusted model, 2 variables were significantly associated with postmeeting surrogate misunderstanding: premeeting misunderstanding (β = 0.30; 95% CI, 0.17-0.44; *P* < .001) and surrogate health literacy (β = 1.50; 95% CI, 0.09-2.98; *P* = .04). eFigure 4A in Supplement 1 demonstrates the flow diagram of cases included in the analysis of postmeeting surrogate misunderstanding. In sensitivity analyses, there were no significant associations between either SDM information or SDM prognosis and surrogate prognostic misunderstanding (SDM information: β = −3.96; 95% CI, −8.29-0.38; *P* = .07; SDM prognosis: β = 1.21; 95% CI, −2.50-4.92, *P* = .52) (Figure 4).

In the subgroup analysis of 78 surrogates with clinically significant premeeting prognostic misunderstanding, there was a statistically significant association between SDM-aligned communication and surrogate misunderstanding (β = −1.71; 95% CI, −3.09 to −0.34; *P* = .01). For every additional physician SDM-aligned communication behavior, surrogate misunderstanding significantly decreased by 1.71 percentage points (Figure 4; eFigure 5 in Supplement 1).

SDM-aligned communication was not significantly associated with physician prognostic misunderstanding in unadjusted (β = 0.31; 95% CI, −0.88-1.50; *P* = .51) and adjusted (β = −0.51; 95% CI, −1.64-0.62; *P* = .38) analyses (Figure 4). In the unadjusted model, every additional SDM-aligned communication behavior was associated with a 0.31–percentage point increase in physician misunderstanding; in the adjusted model, every additional SDM-aligned communication behavior was associated with a 0.51–percentage point decrease in physician misunderstanding, but these associations were not statistically significant.

## Discussion

In this retrospective cohort study, we found that surrogates’ misunderstanding of physicians’ prognostic estimates, though not physicians’ misunderstanding of surrogates’ prognostic estimates, significantly decreased after ICU family meetings. Among all surrogates, SDM-aligned communication during family meetings was not associated with reduced surrogate misunderstanding of physician prognostic estimates. However, SDM-aligned communication was associated with reduced prognostic misunderstanding among those surrogates who had clinically significant prognostic misunderstanding (≥20 percentage points) prior to the family meeting. Our findings suggest that SDM-aligned communication may not consistently improve mutual understanding of survival prognostic estimates but may reduce misunderstanding among those surrogates with greater misunderstanding at the outset.

Our exploratory subgroup analysis suggests there may be an association between SDM-aligned communication and surrogate misunderstanding for surrogates who start with high levels of misunderstanding of survival prognostic estimates. Specifically, SDM-aligned communication may be more impactful with patients and surrogates who have greater potential to demonstrate improvement in outcomes (such as prognostic misunderstanding). Therefore, future decision-making studies should be enriched for participants who have clinically relevant levels of baseline prognostic misunderstanding. Our results also echo recent work showing an association between SDM in ICU family meetings and higher baseline clinician-family discordance: clinicians may consciously or unconsciously use more SDM-aligned communication behaviors when there is a greater gap between their own prognostic estimate and that of the family’s, with the expectation it will reduce this gap.^27^

Our finding that surrogate misunderstanding decreased following family meetings corroborates prior evidence of the value of structured communication in ICU settings.^28,29,30^ Given that SDM-aligned communication was not associated with the observed reduction in surrogate prognostic misunderstanding in the full cohort, additional qualitative analysis may help identify alternative features of these meetings that led to decreased surrogate prognostic misunderstanding. The high prevalence of surrogate prognostic misunderstanding, with nearly half of the surrogates in our sample misunderstanding physicians’ prognostic estimates by more than 20 percentage points even after family meetings, is also in line with prior work demonstrating that surrogate prognostic misunderstanding is common.^10,11^ More broadly, our study findings join a growing body of evidence that patients and families often do not understand important clinical details that their clinical team assumes they do.^2,10,11,31,32^

While SDM is meant to be bidirectional, prior studies of SDM communication have focused far more on its impact on patients and surrogates than clinicians.^33,34,35^ Similarly, there has been little attention paid to clinicians’ misunderstanding of surrogates as opposed to surrogates’ misunderstanding of clinicians. There is no prior work, to our knowledge, determining clinically meaningful clinician prognostic misunderstanding. However, if we extrapolate from surrogate prognostic misunderstanding that clinician prognostic misunderstanding of 20 percentage points or greater may be clinically important, clinician prognostic misunderstanding was present more than one-third of the time after family meetings in our study. This level of misunderstanding concurs with recent work that physicians may not successfully perceive when families do not understand physicians’ prognostic estimates.^10^ A clinician’s inability to accurately perceive how a surrogate understands the information they convey may limit opportunities to improve surrogate misunderstanding. Overall, clinicians’ ability to perceive patients and surrogates accurately is an important skill in clinical interactions that is included in multiple models of communication in health care and worthy of further study.^36^

Identifying communication practices that have a causal relationship with accurate surrogate perception of clinician prognostic estimates, and the converse, is also a fruitful area for further research. Additional qualitative analysis is indicated to identify other features of communication in family meetings that are associated with reduced misunderstanding. In addition, future work assessing prognostic misunderstanding should explore communication that occurs outside of family meetings, such as that which occurs during rounds or in unstructured, routine interactions during care delivery. Overall, continued study of the overlap (or lack thereof) in patient, family, and clinician perception of the other party’s beliefs may provide important insights in improving patient-family-clinician communication and better delivery of value-concordant care to seriously ill patients.^32,44^

It is worth noting that our measure of SDM-aligned communication differs from other measures of SDM. There is no gold-standard measure of SDM in the ICU, corresponding to ongoing debate on what exactly SDM is, what it is not, and how to measure it, especially in the context of the ICU where the conduct and content of SDM may substantially differ from other settings given the time-sensitive, high-stakes nature of decisions in the ICU.^16,37,38,39,40,41,42^ Strengths of our measure include (1) representation of the 3 core elements of SDM that have been consistently described in SDM conceptual models (ie, information exchange, deliberation, making a decision), (2) its development specifically for use in the setting of critical illness and thus its inclusion of context-specific, decision-relevant communication behaviors, and (3) its prior validation that demonstrated an association with goal-concordant care delivery, the ultimate purpose of SDM.^15^

Our study’s other strengths include its empirical assessment of the association between SDM-aligned communication and prognostic misunderstanding, its use of data from 5 sites, and its distinction between prognostic discordance and prognostic misunderstanding. In addition, its serial assessment of surrogate and physician estimates of survival prognosis and perceptions of the other party’s estimates before and after a family meeting is unique in its ability to implicate causality in the family meeting’s effect on surrogate misunderstanding.^2^

### Limitations

Our study also has limitations. It is possible that we did not detect associations between SDM-aligned communication and surrogate or physician prognostic misunderstanding in the full cohort because we were underpowered to do so. It is also possible that unmeasured physician or surrogate level characteristics biased our results toward the null in our primary analyses. Generalizability of our findings may be limited as the surrogates in our study were family members of incapacitated patients receiving prolonged mechanical ventilation with a potential selection bias toward surrogates with more fixed prognostic expectations (as they have chosen to continue life-sustaining treatments). Additionally, we examined only 1 specific aspect of communication (SDM-aligned communication) and only communication that occurred within a family meeting. While these last 2 limitations were intentional, further work is warranted to more comprehensively identify communication approaches that reduce misunderstanding in the ICU.

## Conclusions

In this retrospective cohort study of 137 family meetings in the ICU, SDM-aligned communication was not associated with changes in prognostic misunderstanding for all surrogates or physicians, but it was associated with reduced prognostic misunderstanding among surrogates with clinically significant misunderstanding at baseline. SDM has been called the “pinnacle of patient-centered care,”^42^ and is considered by many to be the gold standard of preference-sensitive medical decision making.^43^ While it may be assumed that SDM results in mutual understanding of its participants, our multicenter study of recorded ICU family meetings only partially supports this assumption.

## Notes

Supplement 1. **eFigure 1.** Clinician Misunderstanding of Surrogate Prognostic Estimate **eTable.** SDM-Aligned Communication Behaviors **eFigure 2.** Directed Acyclic Graphs **eFigure 3.** Changes in Surrogate Misunderstanding of Physician Estimate of Survival Prognosis Pre– to Post–Family Meeting **eFigure 4. **Postmeeting Misunderstanding Flow Diagrams **eFigure 5.** Scatterplot of SDM-Aligned Communication and Postmeeting Surrogate Misunderstanding, by Level of Premeeting Misunderstanding
eReferences.

Supplement 2.
Data Sharing Statement
