# Supplementary material for: Shared Decision-Making Communication and Prognostic Misunderstanding in the ICU
Source: JAMA Netw Open. 2024 Oct 15;7(10):e2439715. doi: 10.1001/jamanetworkopen.2024.39715 (PMC11581528; doi:10.1001/jamanetworkopen.2024.39715)
Supplement: Supplement 1. — eFigure 1. Clinician Misunderstanding of Surrogate Prognostic Estimate eTable. SDM-Aligned Communication Behaviors eFigure 2. Directed Acyclic Graphs eFigure 3. Changes in Surrogate Misunderstanding of Physician Estimate of Survival Prognosis Pre– to Post–Family Meeting eFigure 4. Postmeeting Misunderstanding Flow Diagrams eFigure 5. Scatterplot of SDM-Aligned Communication and Postmeeting Surrogate Misunderstanding, by Level of Premeeting Misunderstanding eReferences. [file jamanetwopen-e2439715-s001.pdf]

Supplemental Online Content

Vick JB, Berger BT, Ubel PA, et al. Shared decision-making communication and prognostic misunderstanding in the ICU. *JAMA Netw Open.* 2024;7(10):e2439715. doi:10.1001/jamanetworkopen.2024.39715

eFigure 1. Clinician Misunderstanding of Surrogate Prognostic Estimate

eTable. SDM-Aligned Communication Behaviors

eFigure 2. Directed Acyclic Graphs

eFigure 3. Changes in Surrogate Misunderstanding of Physician Estimate of Survival Prognosis Pre– to Post–Family Meeting

eFigure 4. Postmeeting Misunderstanding Flow Diagrams

eFigure 5. Scatterplot of SDM-Aligned Communication and Postmeeting Surrogate Misunderstanding, by Level of Premeeting Misunderstanding

eReferences.

This supplemental material has been provided by the authors to give readers additional information about their work.

eFigure 1. Clinician Misunderstanding of Surrogate Prognostic Estimate

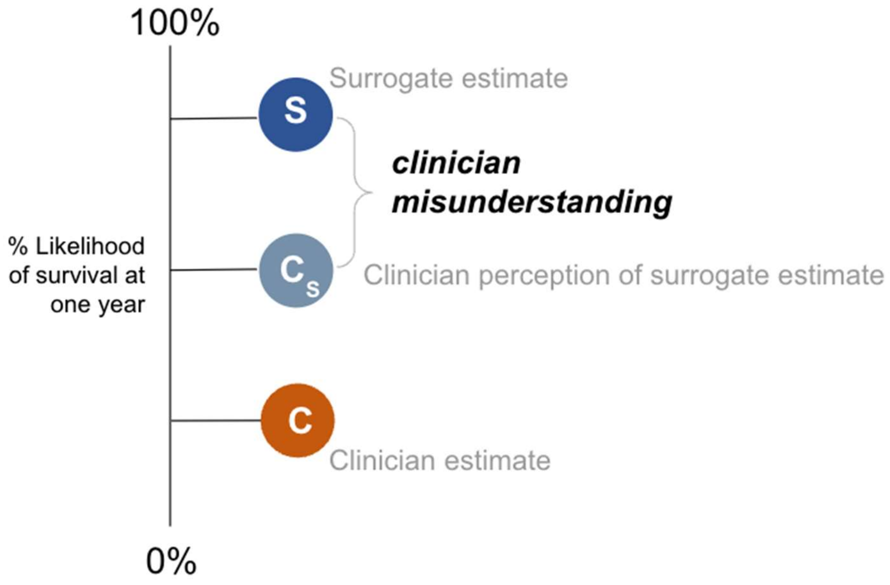

This figure illustrates the definition of clinician misunderstanding, which is the absolute value of the difference between the surrogate's estimate of a patient's prognosis and the clinician's perception of the surrogate's estimate. For example, a surrogate might estimate that a patient has a 90% change of survival at one year. A clinician might think that the surrogate thinks that the patient has a 50% chance of survival. Clinician misunderstanding in this example would then be 40%.

Please note that this figure is illustrative only and not based on the data included in our analysis.

S: Surrogate prognostic estimate. C_S: Clinician perception of surrogate prognostic estimate. C: Clinician prognostic estimate. Clinician misunderstanding of surrogate prognostic estimate is an absolute value: $|S-C_S|$

eTable. SDM-Aligned Communication Behaviors

Component	*ACCM/ATS SDM Element*
Providing the purpose of the visit	Information exchange
Using the term "death" or similarly explicit terminology	Information exchange
Asking what the family knows about the critical illness	Information exchange
Discussing prognosis, including for survival, functional capacity, length of recovery, or discharge disposition	Information exchange
Requesting the family's permission to proceed or share information	Deliberation
Eliciting preferences, values, goals	Deliberation
Explicitly telling the family that they have made a good decision/are doing a good job	Deliberation
Asking if the family wants others involved in further discussions	Deliberation
Asking if the family wants spiritual support	Deliberation
Linking treatment recommendations to stated preferences	Make a treatment decision
Responding to stated preferences by offering an alternative, restating, or further exploring preferences	Make a treatment decision
Asking if the family has questions about the plan	Make a treatment decision
Checking for agreement with the plan	Make a treatment decision

While there is no well-validated observer-based tool of SDM in the ICU, the elements of our measure of SDM-aligned communication overlap with the three essential elements of SDM in the ICU according to the a consensus statement published by the American College of Critical Care Medicine (ACCM) and the American Thoracic Society (ATS): information exchange, deliberation, and making a treatment decision.[1]

The behavior "using only euphemisms for death" was coded our earlier secondary analysis but was not identified as an SDM-aligned behavior as in the prior simulation study given that it is considered a negative communication behavior. [2,3]

© 2024 Vick JB et al. *JAMA Network Open.*

eFigure 2. Directed Acyclic Graphs

eFigure 2a: Directed Acyclic Graph of Surrogate Misunderstanding of Physician Prognostic Estimate

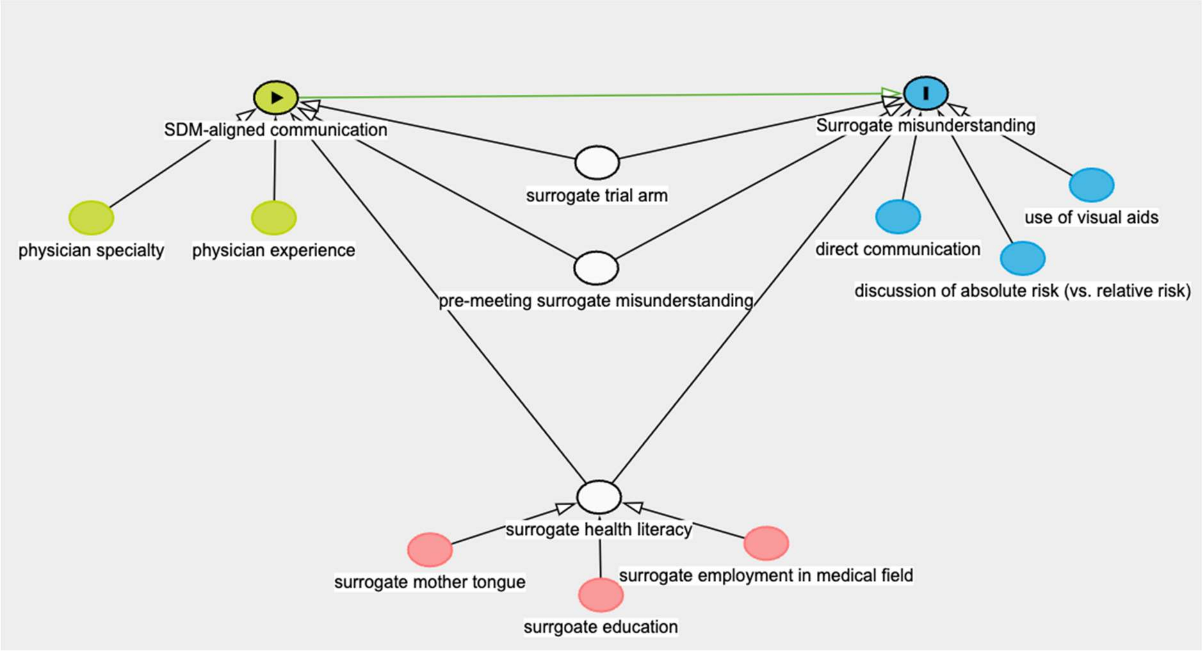

This directed acyclic graph (DAG), created in DAGitty, illustrates the conceptually-related confounders included in our adjusted analysis of SDM-aligned communication and post-meeting surrogate misunderstanding: pre-meeting surrogate misunderstanding, surrogate trial arm, and surrogate health literacy.

eFigure 2b: Directed Acyclic Graph of Physician Misunderstanding of Surrogate Prognostic Estimate

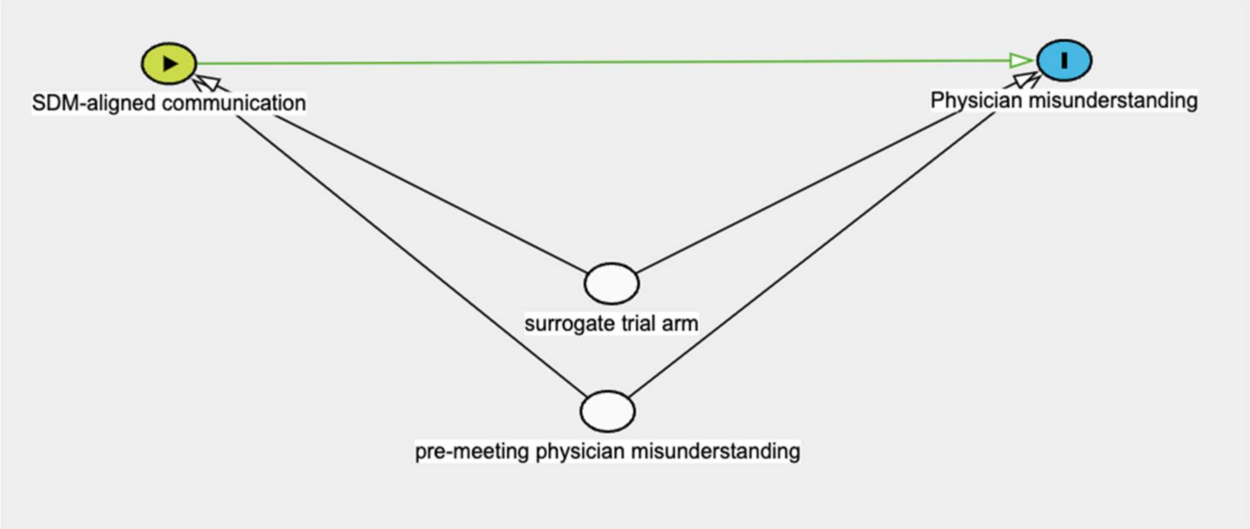

This directed acyclic graph (DAG) created in DAGitty, illustrates the conceptually-related confounders included in our adjusted analysis of SDM-aligned communication and post-meeting physician misunderstanding: pre-meeting physician misunderstanding and surrogate trial arm.

© 2024 Vick JB et al. *JAMA Network Open.*

eFigure 3. Changes in Surrogate Misunderstanding of Physician Estimate of Survival Prognosis Pre– to Post–Family Meeting

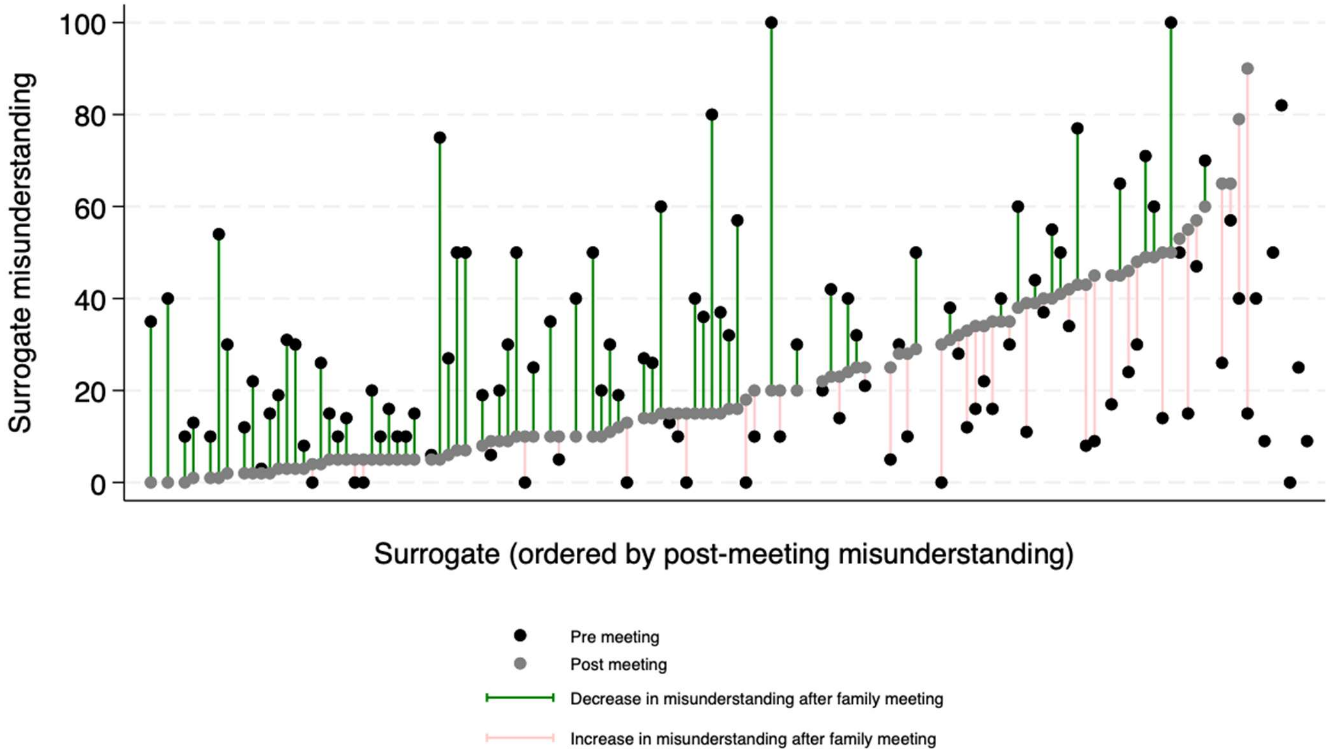

For each surrogate, the distance between each black dot (pre-meeting surrogate misunderstanding) and gray dot (post-meeting misunderstanding) represents the difference in prognostic misunderstanding from before to after the family meeting. A greater number of surrogates had a decrease in their prognostic misunderstanding (green lines) than increase in prognostic misunderstanding (light red lines).

© 2024 Vick JB et al. *JAMA Network Open.*

eFigure 4. Postmeeting Misunderstanding Flow Diagrams

eFigure 4a: Post-meeting surrogate misunderstanding flow diagram

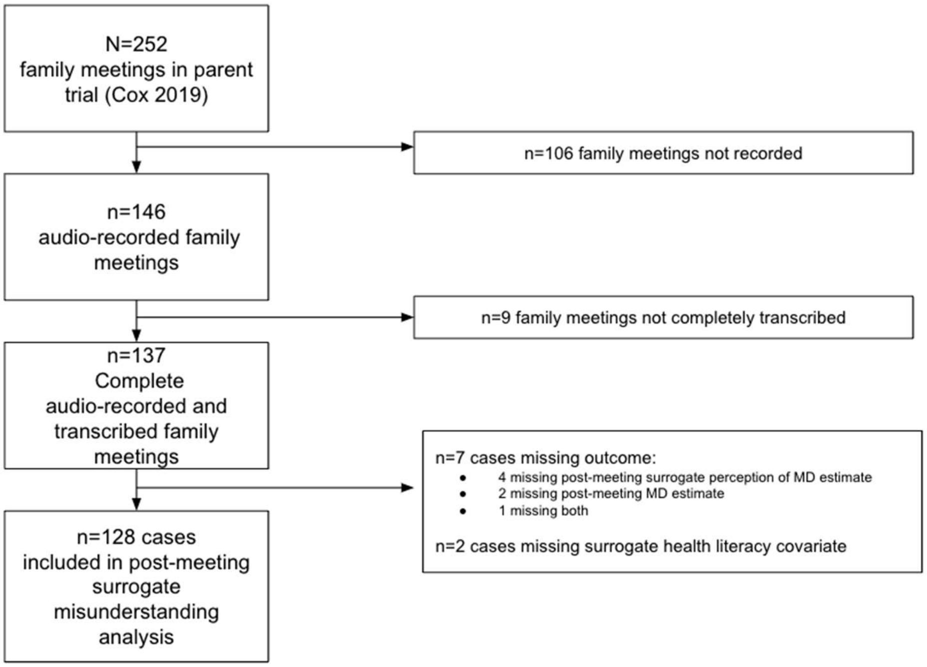

This flow diagram explains the difference between the number of family meetings that occurred with primary surrogates during the parent trial (N=252) and the number of family meetings included in the analysis of the association between SDM-aligned communication and post-meeting surrogate misunderstanding (n=128).[4]

eFigure 4b: Post-meeting physician misunderstanding flow diagram

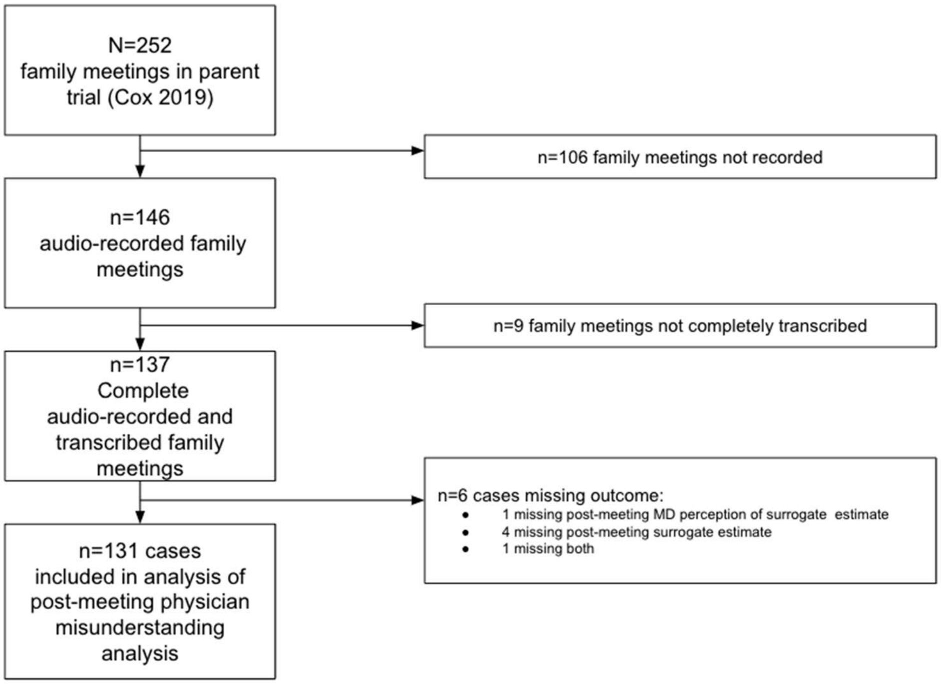

This flow diagram explains the difference between the number of family meetings that occurred with primary surrogates during the parent trial (N=252) and the number of family meetings included in the analysis of the association between SDM-aligned communication and post-meeting surrogate misunderstanding (n=131). [4]

© 2024 Vick JB et al. *JAMA Network Open.*

eFigure 5. Scatterplot of SDM-Aligned Communication and Postmeeting Surrogate Misunderstanding, by Level of Premeeting Misunderstanding

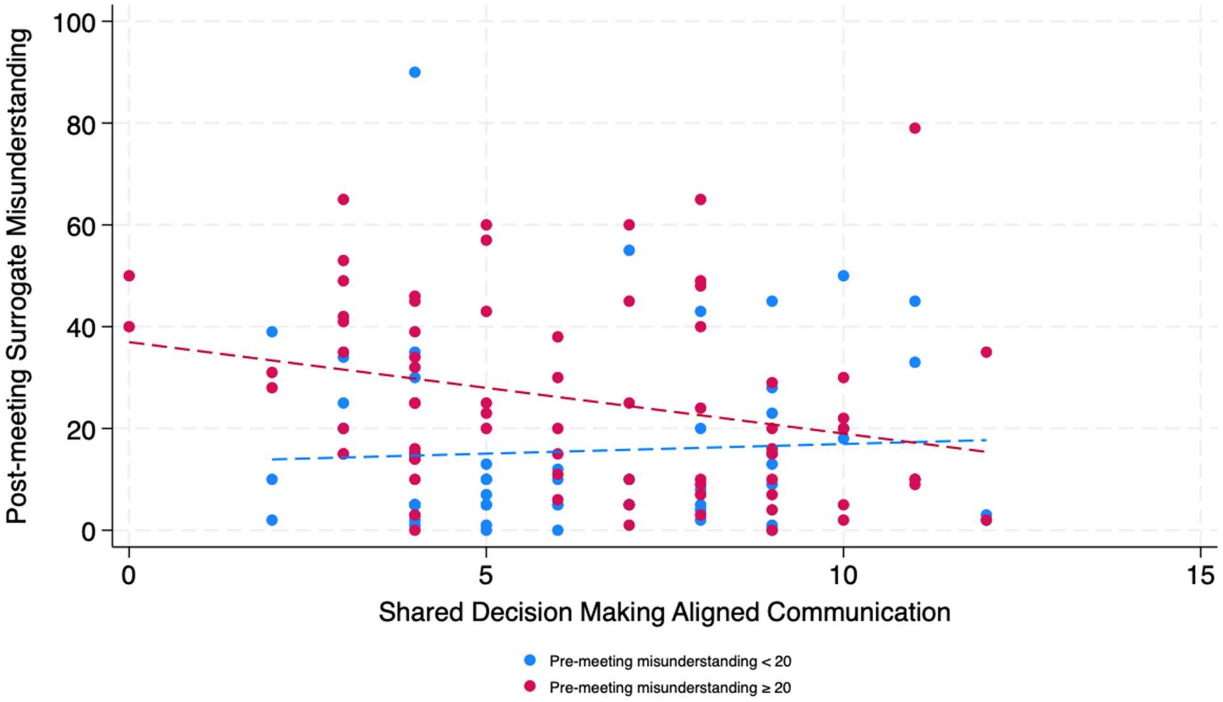

Among surrogates with prognostic misunderstanding of at least 20 percentage points (red dots), post-meeting misunderstanding decreased as SDM-aligned communication decreased. This relationship was not seen among those surrogates with pre-meeting misunderstanding less than 20 (blue dots).

© 2024 Vick JB et al. *JAMA Network Open.*

eReferences

1 Kon, A. A. *et al.* Shared Decision Making in ICUs: An American College of Critical Care Medicine and American Thoracic Society Policy Statement. *Crit Care Med* **44**, 188-201 (2016). https://doi.org:10.1097/CCM.0000000000001396

2 You, H. *et al.* Racial Differences in Physicians' Shared Decision-making Behaviors during Intensive Care Unit Family Meetings. *Ann Am Thorac Soc* (2023). https://doi.org:10.1513/AnnalsATS.202212-997RL

3 Mohan, D., Alexander, S. C., Garrigues, S. K., Arnold, R. M. & Barnato, A. E. Communication practices in physician decision-making for an unstable critically ill patient with end-stage cancer. *J Palliat Med* **13**, 949-956 (2010). https://doi.org:10.1089/jpm.2010.0053

4 Cox, C. E. *et al.* Effects of a Personalized Web-Based Decision Aid for Surrogate Decision Makers of Patients With Prolonged Mechanical Ventilation: A Randomized Clinical Trial. *Ann Intern Med* **170**, 285-297 (2019). https://doi.org:10.7326/M18-2335

© 2024 Vick JB et al. *JAMA Network Open.*
