# Supplementary material for: Shared Decision-Making Communication and Prognostic Misunderstanding in the ICU
Source: JAMA Netw Open. 2024 Oct 15;7(10):e2439715. doi: 10.1001/jamanetworkopen.2024.39715 (PMC11581528; doi:10.1001/jamanetworkopen.2024.39715)
Supplement: Supplement 2. — Data Sharing Statement [file jamanetwopen-e2439715-s002.pdf]

Data Sharing Statement

Vick. Shared Decision-Making Communication and Prognostic Misunderstanding in the ICU. *JAMA Netw Open*. Published October 15, 2024. doi:10.1001/jamanetworkopen.2024.39715

Data
Data available: Yes
Data types: Deidentified participant data
How to access data: Deidentified participant data accessible to researchers whose proposed use of the data has been approved for any purpose approved by the study authors and Duke University Health System IRB after approval of a protocol and signed data access agreement.
When available: With publication

Supporting Documents
Document types: Statistical/analytic code
How to access documents: Statistical/analytic code will be available from the corresponding author on request (judith.vick@duke.edu).
When available: With publication

Additional Information
Who can access the data: Deidentified participant data accessible to researchers whose proposed use of the data has been approved for any purpose approved by the study authors and Duke University Health System IRB after approval of a protocol and signed data access agreement.
Types of analyses: Deidentified participant data accessible to researchers whose proposed use of the data has been approved for any purpose approved by the study authors and Duke University Health System IRB after approval of a protocol and signed data access agreement.
Mechanisms of data availability: Deidentified participant data accessible to researchers whose proposed use of the data has been approved for any purpose approved by the study authors and Duke University Health System IRB after approval of a protocol and signed data access agreement.
